# 344 Impact of a far ultraviolet-C light intervention on dissemination of bacteriophage MS2 and a DNA marker from portable medical equipment

**DOI:** 10.1017/ash.2026.10684

**Published:** 2026-06-23

**Authors:** Grace St. Cyr, Devin Ray, Linda Leach, Logan Grimes

**Affiliations:** 1 The Children’s Hospital of Philadelphia; 2 Children’s Hospital of Philadelphia

## Abstract

**Background:** Antibiotic-resistant gram-negative (RGN) bacteria pose a significant threat to child health, leading to use of contact precautions to limit nosocomial transmission. While adult data increasingly support selective discontinuation of precautions, pediatric-specific guidance remains limited due to uncertainty around the duration of RGN colonization. Based on institutional expert opinion, on November 19th, 2021, our institution implemented a policy allowing removal of contact precautions after one year for patients without subsequent RGN culture positivity. This study aimed to characterize RGN carriage duration, identify risk factors for prolonged carriage, and evaluate the impact of the policy change on hospital-associated RGN incidence. **Methods:** We conducted a retrospective chart review of patients ages 0-21 years with identified RGNs in the hospital electronic medical record system from May 2021 to November 2025. RGNs of interest included extended-spectrum-beta-lactamase (ESBL) Enterobacteriaceae along with multidrug-resistant (MDR) Enterobacteriaceae, Pseudomonas aeruginosa, and Acinetobacter species. Patients with carbapenem-resistant isolates or cystic fibrosis were excluded based on ineligibility for removal of precautions. Data extraction focused on each patient’s first documented RGN infection during the queried timeframe including organism, resistance pattern, and specimen source. Additional evaluation included whether the isolate was treated as an active infection, subsequent positivity with any RGN isolate, and potential risk factors for recurrent RGN positivity. Categorical exposure variables were assessed via ?² test of association and Wilcox ranked sum test to determine differences in duration of RGN carriage (R v4.5.1). **Results:** We identified 232 patients with RGN isolates. Of these, 158 met inclusion criteria with most exclusions (53/232) due to carbapenem-resistance in the initial RGN isolate. Most patients (91/158, 57.6%) did not have subsequent positivity with any RGN. Of those with durable carriage of the same RGN species (49/158, 31.0%) the median was 269 days (range 39 – 1463). Only presence of underlying genitourinary complexity was associated with risk of subsequent positivity with the same RGN (?² = 10.045, p = 0.001527) and prolonged median duration of RGN carriage of 396 days (Wilcox ranked sum test of identical distributions, W = 428, p = 0.01004). When comparing the seven months leading up to and following the policy change implementation period, there was no increase in incident hospital-associated RGN infections per 100,000 inpatient days. Conclusions Current hospital epidemiology supports a policy of removal of contact precautions after one year of no further RGN positivity and may serve to inform broader pediatric policy decisions.

